# Struvite-based composites for slow-release fertilization: a case study in sand

**DOI:** 10.1038/s41598-022-18214-8

**Published:** 2022-08-19

**Authors:** Stella F. Valle, Amanda S. Giroto, Vitalij Dombinov, Ana A. Robles-Aguilar, Nicolai D. Jablonowski, Caue Ribeiro

**Affiliations:** 1 Department of Chemistry, Federal University of São Carlos, Washington Luiz Highway, km 235, São Carlos, SP 13565-905 Brazil; 2 Embrapa Instrumentation, XV de Novembro Street, n 1452, São Carlos, SP 13560-970 Brazil; 3 Institute of Bio- and Geosciences, IBG-2: Plant Sciences, Forschungszentrum Jülich GmbH, 52425 Jülich, Germany; 4 Department of Green Chemistry and Technology, Faculty of Bioscience Engineering, Ghent University, Campus Coupure, Infinity House, Coupure Links 615, 9000 Ghent, Belgium; 5 IRTA Institute of Agrifood Research and Technology, Torre Marimon, E08140 Caldes de Montbui, Barcelona, Spain

**Keywords:** Plant sciences, Polymer chemistry

## Abstract

Struvite (St) recovered from wastewaters is a sustainable option for phosphorus (P) recovery and fertilization, whose solubility is low in water and high in environments characterized by a low pH, such as acidic soils. To broaden the use of struvite in the field, its application as granules is recommended, and thus the way of application should be optimized to control the solubility. In this study struvite slow-release fertilizers were designed by dispersing St particles (25, 50, and 75 wt%) in a biodegradable and hydrophilic matrix of thermoplastic starch (TPS). It was shown that, in citric acid solution (pH = 2), TPS promoted a steadier P-release from St compared to the pure St pattern. In a pH neutral sand, P-diffusion from St-TPS fertilizers was slower than from the positive control of triple superphosphate (TSP). Nevertheless, St-TPS featured comparable maize growth (i.e. plant height, leaf area, and biomass) and similar available P as TSP in sand after 42 days of cultivation. These results indicated that St-TPS slow P release could provide enough P for maize in sand, achieving a desirable agronomic efficiency while also reducing P runoff losses in highly permeable soils.

## Introduction

Phosphorus (P) fertilization plays a crucial role in sustaining the increasing demand for agricultural production, being one of the most limiting nutrients for crop yields and quality^1^. Mined phosphate rocks are currently the main source for manufacturing commercial P fertilizers^2,3^. However, as finite resources, P rock reserves are being depleted, threatening long-term global food security^4–6^. Additionally, the readily soluble conventional mineral fertilizers are prone to runoff losses, contributing to the eutrophication of local water bodies, which significantly impacts the local ecosystem and water quality^6–8^.

Struvite (MgNH_4_PO_4_·6H_2_O) stands out as an alternative to conventional P management, as it tackles both the P scarcity and pollution problems^9–12^. Struvite crystals can be easily obtained by treating urban wastewaters under alkaline conditions, which can be economically feasible in large scale, in typical water treatment stations. Therefore, its production has the benefit of recovering phosphate from waste and preventing it from re-entering the watercourses and damaging the environment^13,14^. As a P fertilizer, struvite provides additional essential nutrients to plant growth, i.e., nitrogen (N) and magnesium (Mg), which can synergistically enhance P offtake in some crops^10^. This alternative P source is considered a slow-release fertilizer due to its low water solubility, which reduces its susceptibility to runoff losses^13,15,16^. On the other hand, struvite-P release rate may be insufficient to meet plants' demands, especially in the early stages of crop development^16^. This situation could be reversed by reducing struvite particle size and thus increasing its dissolution rate^17–20^. However, for field application, fertilizers in granular form are usually preferred for practical and safety reasons^16,17^.

A strategy to control the dissolution pattern of P fertilizers while still offering a granular material is to disperse the ground mineral in a biodegradable matrix, forming a composite^18^. Previously it was found that these matrix-based controlled-release systems can increase P solubility by avoiding particle agglomeration^21–27^. Giroto et al. demonstrated this effect with hydroxyapatite nanoparticles in urea and thermoplastic starch (TPS) hosts^21^. Still, there are some discrepancies in the use of composites. For instance, Valle et al. did not detect faster P release from struvite particles in a similar composite system with a porous polysulfide matrix, which was attributed to a barrier effect from the matrix^27^.

The present study aims to elucidate P release dynamics from sustainable fertilizer composites made of ground struvite embedded in a TPS matrix, using urea as a plasticizer with agronomic value. Thermoplastic starch is a fitting matrix candidate as an easily processable natural polymer obtained from low-cost, non-toxic, and renewable feedstocks^28,29^. We aim to understand whether the hydrophilic nature of TPS would improve struvite dissolution, similarly to hydroxyapatite-TPS composites^21^, or whether TPS would restrict rapid P release from struvite as in the polysulfide matrix^27^. To do that, we investigated (i) P release from fertilizers under laboratory conditions and (ii) effects of P fertilization on maize (*Zea mays*) growth under greenhouse conditions in a model substrate, i.e., a sand with low P sorption capacity, no constituted fertility, and an assumed low microbial activity^30^.

## Results and discussion

### Characterization of the composite materials

**Figure 1:**
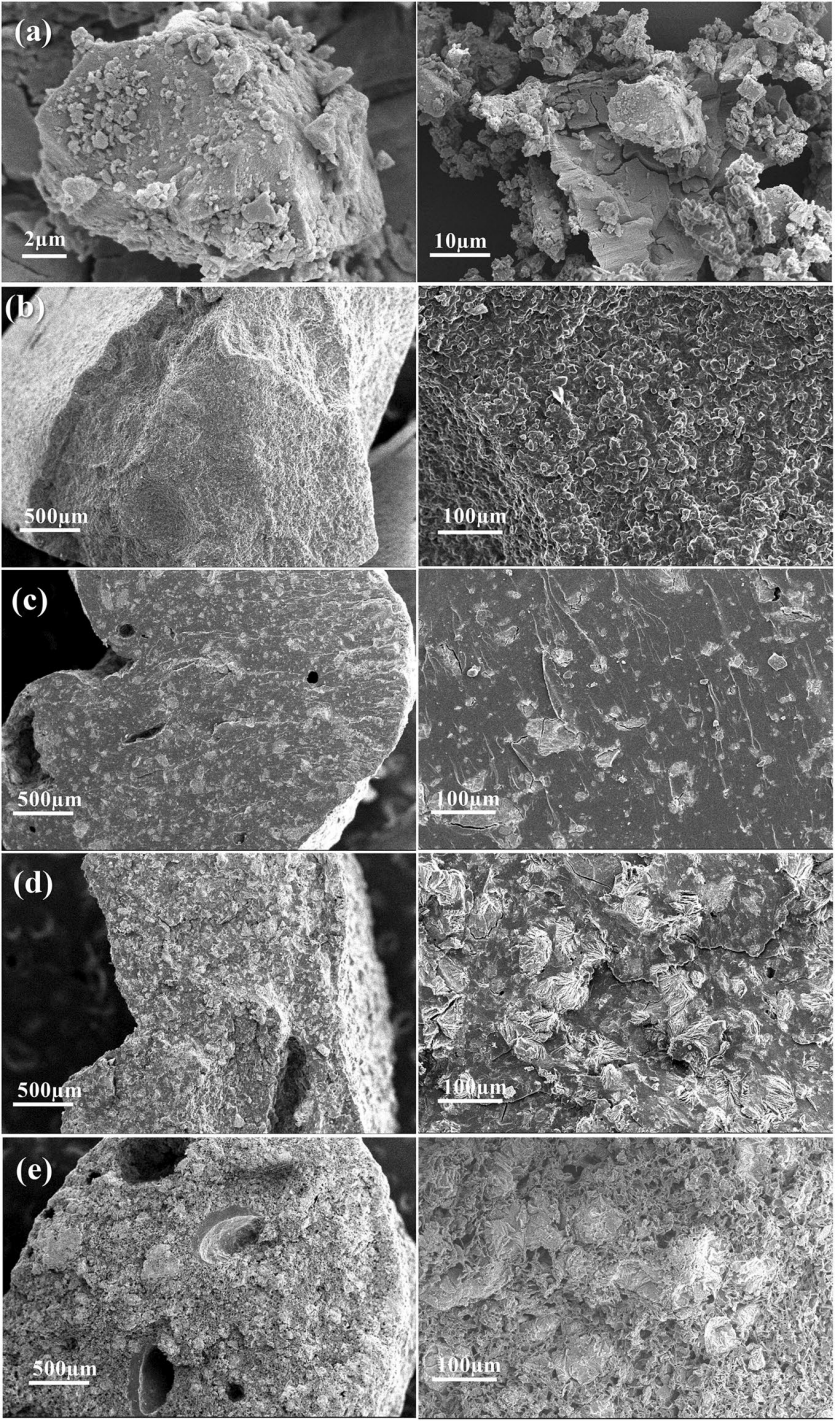
SEM images of (**a**) pure struvite, (**b**) TPS, and composites (**c**) 25 St-TPS, (**d**) 50 St-TPS, and (**e**) 75 St-TPS.

Fertilizers consisting of ground struvite (St) mixed in a thermoplastic starch matrix (TPS) were prepared as alternatives for P supply, using either 25, 50, or 75 wt% of St. Urea was incorporated in the formulation as a plasticizer to TPS structure, balancing the final N percentage of the composites. To elucidate the morphology of St-TPS composites and to confirm that struvite was homogeneously dispersed in the TPS matrix, SEM analyses were conducted (Fig. 1). Pure struvite consists of crystalline particles with irregular shapes (Fig. 1a), as previously reported by Rahman et al.^11^. TPS displays a homogenous material with no porosity or phase separation (Fig. 1b), confirming the complete incorporation of urea within thermoplastic starch structure as a plasticizer. SEM images of the St-TPS composites indicate a uniform distribution of struvite particles over the TPS matrix (Fig. 1c–e). 25St-TPS features struvite domains dispersed in a continuous material (Fig. 1c). As struvite content increased in 50St-TPS, the surface became less smooth (Fig. 1d). Nevertheless, the two phases continued to show great compatibility and adhesion, with no observable vacancies between their surfaces. Struvite and TPS appear to be more intercalated in 75St-TPS (Fig. 1e). Moreover, a vastly porous structure is verified in 75St-TPS, possibly formed during the composite preparation due to the evaporation of water from TPS in the drying step, or even to ammonia and structural water loss from struvite. This porous network could favor struvite dissolution, as it increases the composite surface area and its accessibility to water, increasing struvite interaction with soil solution and root exudates.

**Figure 2:**
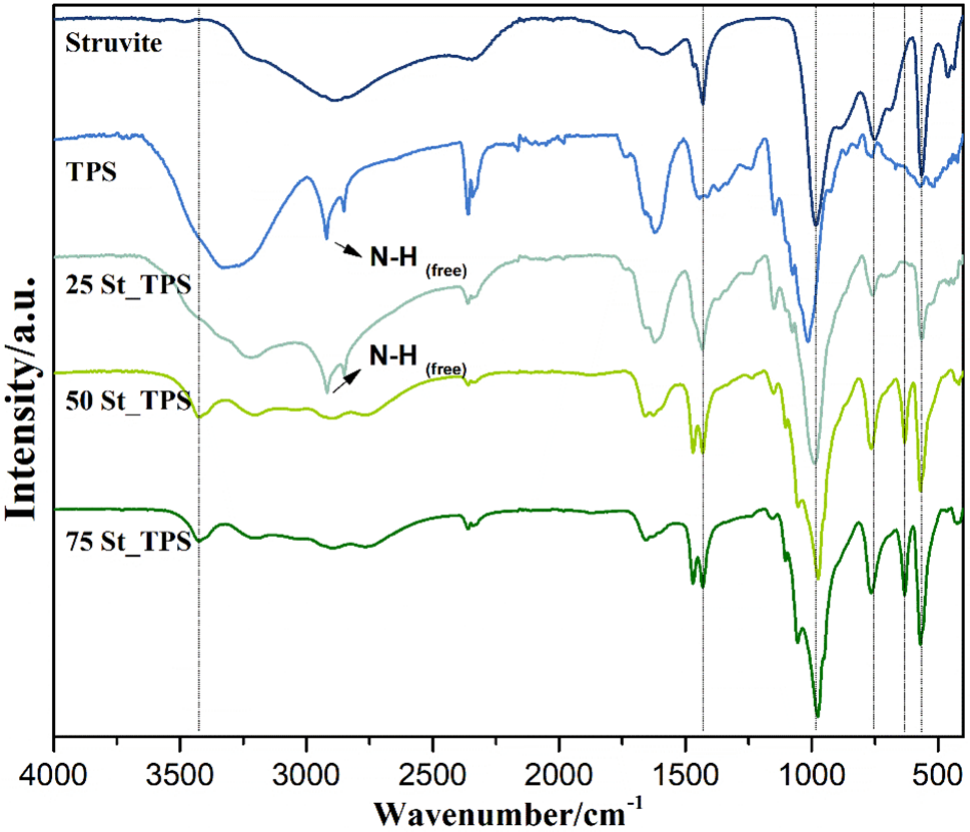
Normalized FTIR spectra of pure struvite, TPS, and composites 25 St-TPS, 50 St-TPS, and 75 St-TPS.

FTIR spectra was analyzed to verify possible changes in the chemical structures of struvite and TPS resulting from the preparation of the composites (Fig. 2). In struvite spectrum (Fig. 2), the region between 3415 and 2098 cm^−1^ corresponds to H–O-H and ammonium N–H stretching vibrations. Pure TPS (Fig. 2) presents a broad band from 3657 to 3000 cm^−1^ related to the stretching of its crystallization water, while typical N–H stretching from urea and NH_4_^+^ units appear as a sharp band at 2920 cm^−1^. The bending mode of water molecules in TPS is also verified (1620 cm^−1^), and N–H bending signal is observed in both struvite and TPS, respectively at 1431 cm^−1^ and 1448 cm^−1^. Struvite features characteristic strong bands from PO_4_ symmetric stretching at 984 cm^−1^ and bending at 565 cm^−1^, in addition to P–O–P stretching at 754 cm^−1^^31^. A weak signal from struvite Mg-O stretching can be found at 461 cm^−1^.

Composite 25St-TPS spectrum shows similarities to both struvite and TPS patterns (Fig. 2). In contrast, 50St-TPS and 75St-TPS present clear distinctions, with the suppression of some struvite bands and the appearance of new signals (Fig. 2), indicating a phase transition from the phosphate crystalline structure. These changes are consistent to the patterns from the dittmarite phase (Mg(NH_4_)(PO_4_)·H_2_O)^32–34^, evidencing the loss of structural water as a result of the temperature used during the preparation of the materials. This modification was reported to occur mostly when struvite is boiled in excess water, similar to starch gelification^11^. Valle et al. (2021) also observed dittmarite formation in the preparation of struvite-polysulfide composite fertilizers^27^, noting that this does not affect the fertilizer efficiency as dittmarite presents a similar P release profile to struvite and higher nutrient concentration^35^. In 25St-TPS, this conversion to dittmarite was probably prevented or lower due to a higher starch content hindering struvite particles. Based on 75St-TPS spectrum, it is possible to verify that structural water loss from struvite could have contributed to the composite porosity (Fig. 1), while ammonium loss did not occur.

While a broad band from 3657 to 3043 cm^−1^ can be seen in 25St-TPS, consistent with O–H and N–H stretching from struvite and TPS, the other composites present a narrower band at 3425 cm^−1^ from dittmarite H_2_O stretching, and typical dittmarite NH_4_ stretching at 3205 cm^−1^ and 2769 cm^−1^ (Fig. 2). Other evidences from dittmarite presence in 50St-TPS and 75St-TPS are the H_2_O bending at 1655 cm^−1^, PO_4_ asymmetric stretching at 1055 cm^−1^, the dislocation of PO_4_ symmetric stretching band to 975 cm^−1^, and the appearance of Mg–O stretching at 632 cm^−1^ (Fig. 2).

### Thermoplastic starch effect on P release in acid solution

**Figure 3:**
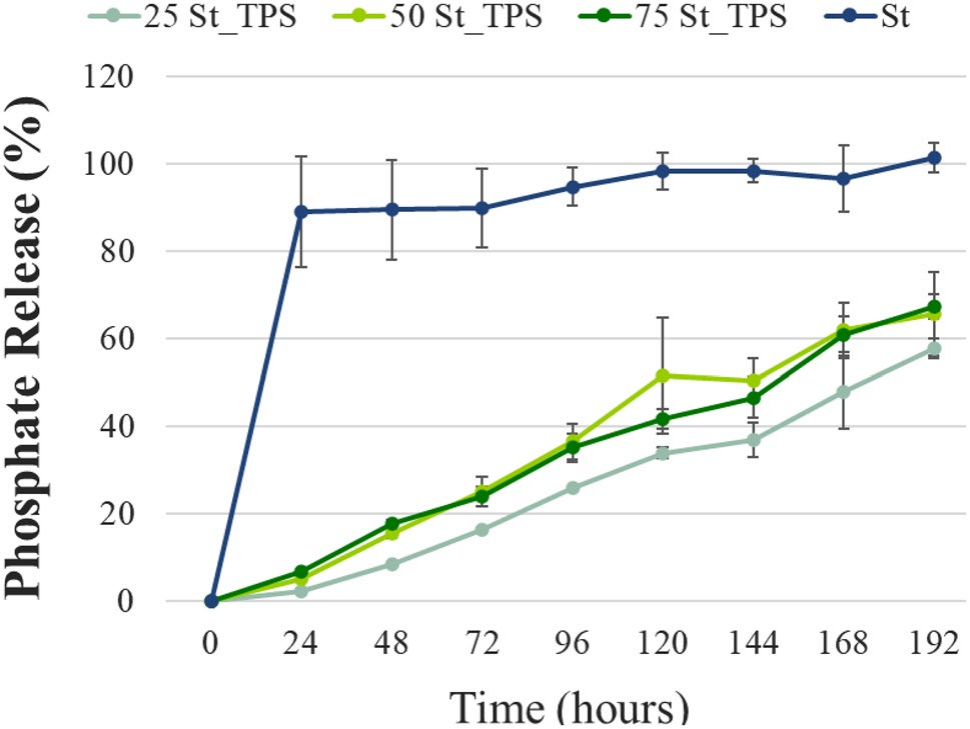
Phosphate release trends in citric acid solution (2 wt%) at 25 °C and pH 2. Points show mean values ± standard deviations (n = 3).

Once the morphological and chemical characteristics of the composites were elucidated, their effect on struvite-P dissolution rate was analyzed. Phosphate release from the composites and pure struvite were measured over time in 2 wt% citric acid solution at initial pH 2 (Fig. 3). This standard test simulates the pattern of phosphate solubilization in a soil–plant system but in a shorter time^36^. Urea release patterns were simultaneously monitored, and discussed in the supplementary information (Fig. S1**)**.

A fast solubilization of pure struvite was observed, with almost 90% of phosphate release in the first 24 h and complete release taking around 120 h (Fig. 3). Struvite dissolution is significantly affected by the pH, being greatly enhanced in acidic environments and reduced under neutral or alkaline conditions^11,16,17^. A slower phosphate release behavior was observed for the composites, suggesting that the TPS matrix functions as a physical barrier to P release. After 192 h, phosphate release was 66% for both 75St-TPS and 50St-TPS, and 55% for 25St-TPS (Fig. 3). Interestingly, composites 75St-TPS and 50St-TPS displayed similar profiles over the experimental period (Fig. 3), despite 75St-TPS having a higher porosity and lower TPS content. 25St-TPS, on the other hand, featured a slower dissolution rate, possibly due to struvite surface being more effectively hindered from the acid solution by a higher polymeric fraction.

Phosphate release from composites is mediated by two processes: particle solubilization and nutrient diffusion. While the matrix can improve the first process with the dispersion effect, it can simultaneously limit the latter. In Giroto et al. (2015), hydroxyapatite nanoparticles in TPS displayed a faster P release compared to the pure mineral, with the matrix mainly acting as a dispersing medium to prevent particle agglomeration^21^. In Valle et al. (2021), a polysulfide matrix reduced struvite-phosphate release rate in acid solution, while P release from Bayóvar rock was enhanced in the same matrix^27^. The differences in the release behavior could be explained by the P source solubility. Since struvite is already readily soluble in acid, the matrix contribution to the solubilization process is not significant, acting instead as a barrier to the fast diffusion. In contrast, apatite solubility is low even in acidic environments, being more affected by the matrix dispersing effect and porosity. In a neutral or alkaline medium, where struvite solubility is low, it is possible that struvite could benefit from this matrix effect on solubilization.

It is worth mentioning that the observed effect of TPS in physically regulating struvite fast solubilization and release in acid medium could be convenient to prevent nutrient loss in soils with low pH or low water retention. Besides TPS barrier effect, other factors could have contributed to a slower P release rate. For instance, struvite-P may chemically interact with the thermoplastic starch chains^21^. Moreover, TPS swelling over time can modify the matrix structure, limiting water and nutrient transportation^21,37^. Urea molecules released from the TPS structure can also have a role in lowering phosphate availability, as they tend to form large complexes with ion species in solution^38,39^. It is essential to highlight that, even though part of struvite was converted to dittmarite during the preparation of the composites (Fig. 2), phosphate solubilization is not affected by this transformation. Massey et al. (2009) found that dittmarite tends to quickly re-hydrate to struvite when in solution, thus displaying an equivalent dissolution to struvite after that^35^.

### Phosphate diffusion from struvite-thermoplastic starch composites in highly permeable sand

**Figure 4:**
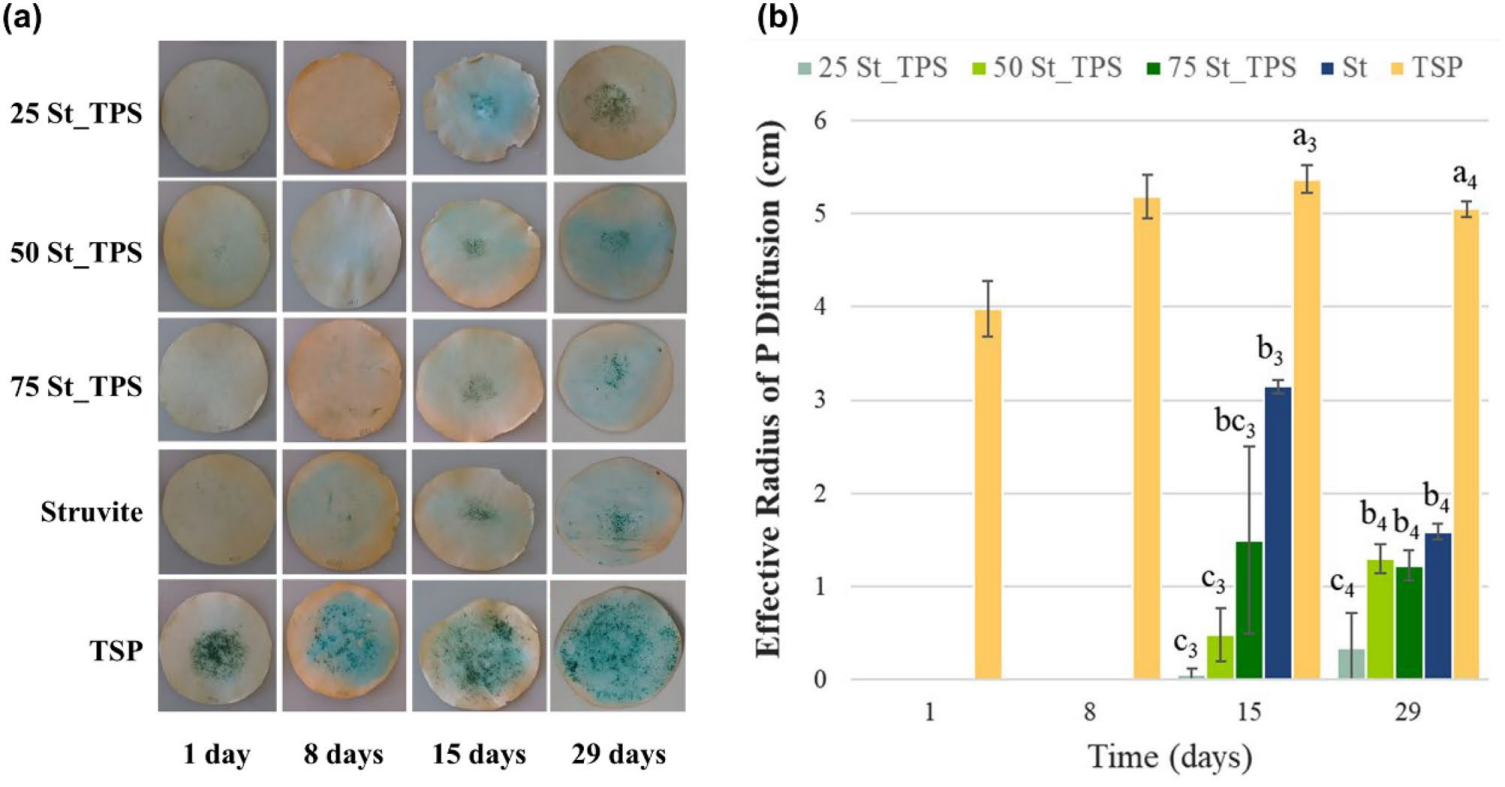
(**a**) P diffusion zone in sand substrate visualized at 1, 8, 15, and 29 days after fertilizer application of St, TSP, and St-TPS composites. (**b**) Effective radius of P diffusion zone (cm) over the incubation time. Bars show mean values ± standard deviations (n = 3). Indexes a, b, and c represent the statistical differences between treatments (*p* < 0.05) for each time group (represented by index numbers).

To simulate the performance of St-TPS fertilizers in degraded sandy soils with low fertility, P diffusion was assessed in sand filled Petri dishes (Fig. 4). The sand substrate was selected due to its low organic matter and clay contents, as well as low concentrations of Fe and Al, which reduce P immobilization processes that could influence the results. Moreover, we were interested in evaluating if the TPS matrix could improve struvite-P solubilization with the particle dispersing effect in a neutral condition, where struvite solubility is lower.

The rapid P diffusion of triple superphosphate (TSP) can be clearly distinguished from the slow-release of struvite and the composites (Fig. 4). As an acidified and highly soluble phosphate source, TSP probably achieved complete dissolution within 8 days, after which the P zone radius stabilized. Despite phosphate’s tendency to retrograde to P-Ca precipitates, the result suggests this effect was minimum^40^. The sand’s high permeability and low sorption capacity allowed a high P mobility to P-unsaturated zones.

Struvite diffusion had a slow initial response, with an effective radius of P diffusion being observed only after 15 days of incubation (Fig. 4). Contrary to TSP, it is highly likely that struvite was not fully solubilized by the end of the experiment, and that P would be released for a longer period than from TSP. Struvite-thermoplastic starch composites also delayed P release, especially as the TPS fraction increased (Fig. 4). Nevertheless, both 50St-TPS and 75St-TPS achieved statistically similar results to pure struvite at the end of the incubation. 25St-TPS smaller and constant diffusion area was consistent with the slower P release rate observed in Fig. 3.

Overall, the results did not indicate that struvite dissolution was improved with its dispersion in TPS over the evaluated incubation time. However, the matrix showed a potential in regulating nutrient delivery and preventing nutrient losses in soils with low retention capacity, in contrast to TSP tendency to leaching. It should be noted that a more significant amount of struvite-P could have solubilized within the matrix system and kept hindered, either due to interactions with starch chains or TPS swelling, not being visualized by this method. Therefore, it is possible that the dispersion effect from the matrix could have been masked. Although it was not conclusive by the results, a contribution from TPS in increasing P dissolution would explain 50St-TPS similar performance to 75St-TPS, both in citric acid and in the diffusion test. Composite 50St-TPS has an intermediate ratio of TPS and struvite, being less affected by a barrier effect than 25St-TPS, but more influenced by particle dispersion than 75St-TPS.

### Effect of slow P release from struvite composites on maize cultivation

A greenhouse experiment was conducted to evaluate the agronomic efficiency of St-TPS composites in sand under maize cultivation. We were interested in investigating if the slow-release character of the composites could benefit maize growth. Nutrient release is expected to be controlled mainly by the fertilizer characteristics but, additionally, by its interaction with plant root exudates. A wide variety of organic compounds can be exudated by roots depending on the plant species, including organic acids and carboxylates that promote nutrient mobilization^41–43^.

**Figure 5:**
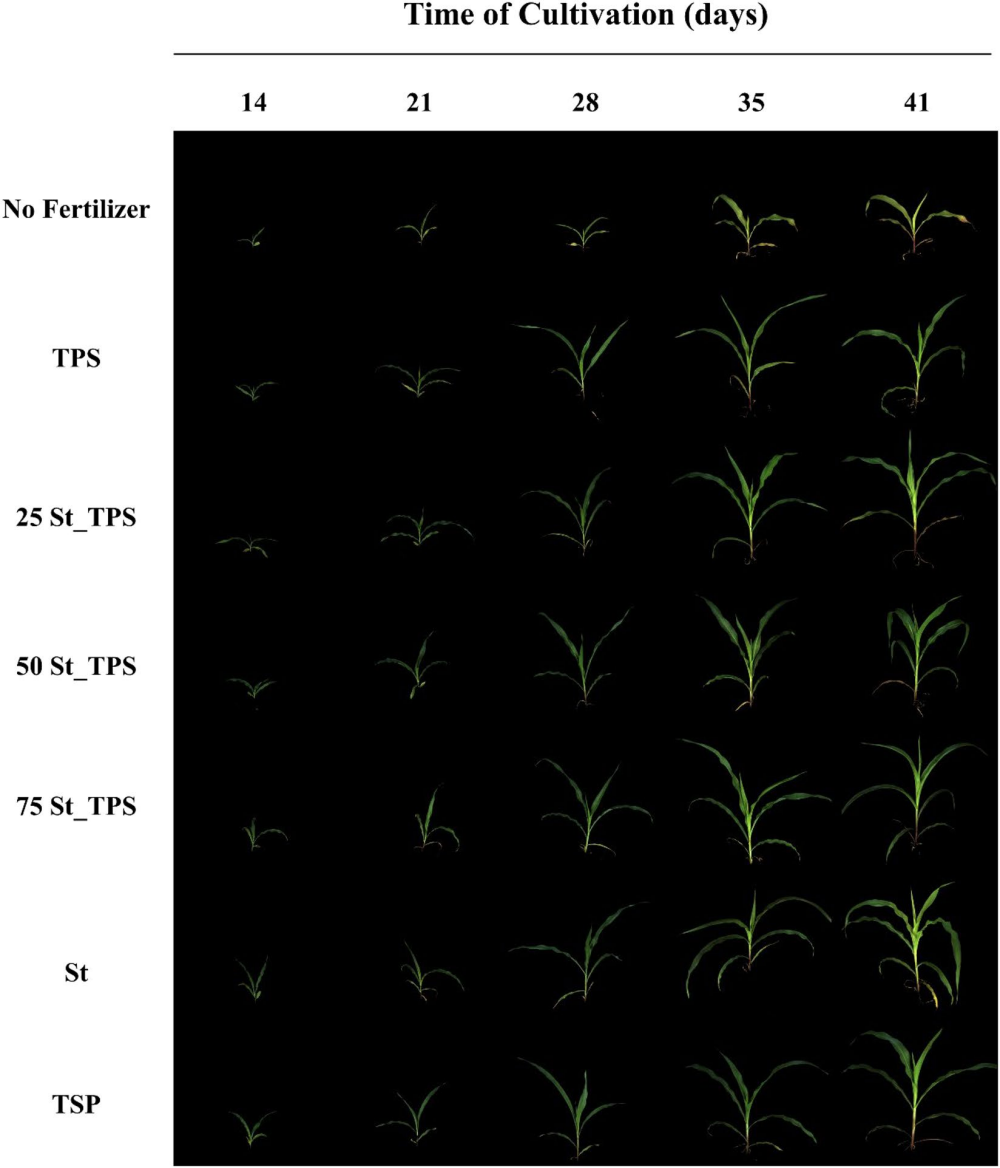
Representative images from maize cultivation over time at a 45° angle, with the no fertilizer control, pure thermoplastic starch (TPS), composites 25 St-TPS, 50 St-TPS, and 75 St-TPS, pure struvite (St), and the positive reference of triple superphosphate (TSP).

Figure 5 shows plant growth under different treatments. Visually, it is possible to notice a superior development from P-fertilized plants, with a similar performance between struvite-based treatments and the positive reference of TSP. Nitrogen deficiency symptoms can be seen in the unfertilized control plant, with the yellowing of older leaves in 35 days of cultivation.

**Figure 6:**
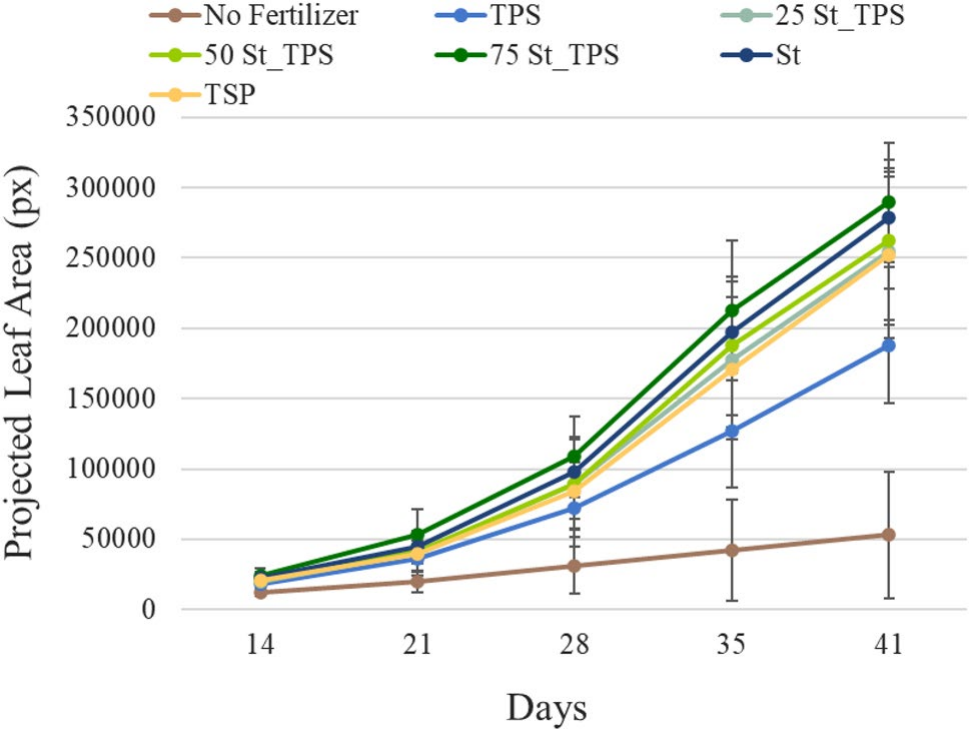
Trends of projected leaf area of each treatment over time. Bars show mean values ± standard deviations (n = 16).

Table S6 and Fig. 6 show the average projected leaf area of each treatment over the period of maize growth, while Table S6 shows additionally the estimated percentage of brown leaf areas. The no fertilizer control plants displayed a significantly slower growth than the others and a proportionally higher brown leaf area, indicating nutrient deficiency. By the end of 41 days, control plants reached 53 × 10^3^ px of projected leaf area, of which 25% was brown (Table S6). The final projected leaf area of plants fertilized with St-TPS composites, St, and TSP was significantly higher compared to no fertilizer control plants and varied between 252 × 10^3^ and 289 × 10^3^ px, while the brown area was around 6% (Table S6). Although the St-TPS composites showed a slower P release than TSP and struvite in the diffusion test (Fig. 4), maize leaf area remained statistically similar between them throughout the greenhouse experiment (Table S6), suggesting enough P was provided to support maize growth in all the developmental stages. Moreover, plants receiving P from struvite-based fertilizers, especially 75St-TPS, generally showed a slightly superior projected leaf area than the TSP reference plants, although the difference was not statistically significant (*p* < 0.05; Table S6).

The projected leaf areas of maize plants fertilized with only TPS were significantly higher than in no fertilizer control plants but lower than in plants fertilized with St, St-TPS, and TSP, with a final value of 187 × 10^3^ px and only 8% brown area at the end of cultivation (Table S6). Interestingly, the projected leaf area in maize plants treated with TPS was statistically similar to that of St, TSP, and St-TPS within the first 28 days, but did not keep up with their growth after that. It is important to highlight that a modified Hoagland solution was applied at 30 days of cultivation only to treatments with St, TSP, and St-TPS composites, providing additional plant nutrients. Thus, compared to these fertilized treatments, the growth of plants under just TPS was probably limited by lower availability of nutrients, displaying a slower trend after 30 days of cultivation (Fig. 6).

**Figure 7:**
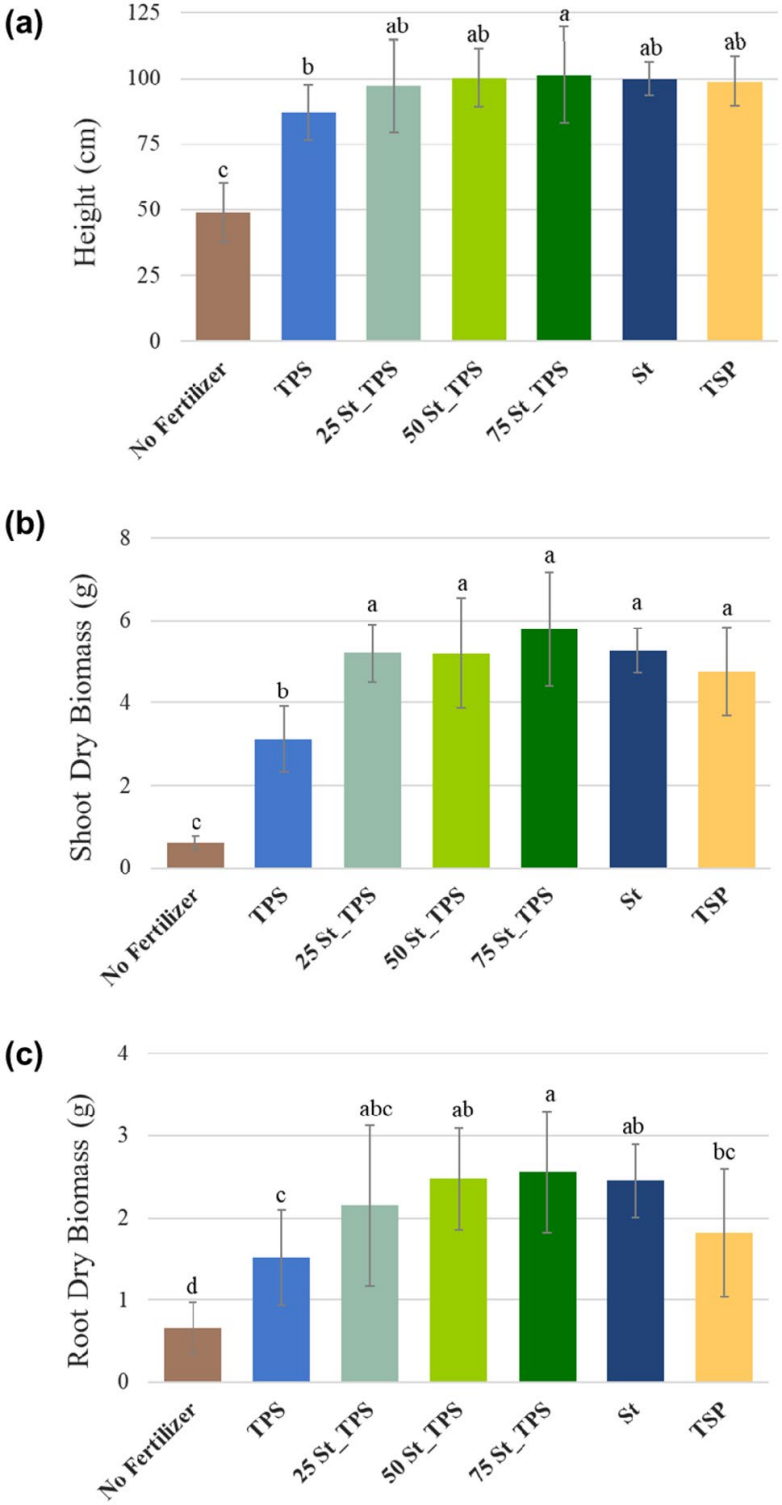
Average (**a**) plant height and dry biomass of (**b**) shoots and (**c**) roots achieved after 42 days of maize cultivation. Bars show mean values ± standard deviations (n = 16 for plant height, n = 15 for shoot biomass, and n = 17 for root biomass). Indexes a, b, c, and d represent the statistical differences between treatments (*p* < 0.05).

In line with projected leaf area measurements, all fertilizer treatments significantly increased the plant height and dry biomass compared to the unfertilized control plants in 42 days (Fig. 7). Shoot:root ratios (Fig. S2) from the P-fertilized treatments were also statistically higher than the no fertilizer control, showing the prominence of shoot production over roots (shoot:root > 2), which could indicate nutrient availability as P deprivation usually leads to a lower shoot:root ratio and to changes in root architecture^44^. Struvite-based fertilizers achieved higher shoot biomass yields than the positive reference (Fig. 7b,c), although not statistically significant, indicating a desirable agronomic efficiency. Interestingly, composite 75St-TPS reached superior root biomass than TSP, with 1.4 times the dry weight (Fig. 7c). In a previous research, soybean plants treated with struvite-polysulfide composites also achieved a higher root production than TSP fertilized plants, with intense growth and distribution of roots in the region of fertilizer application^45^. This was attributed to struvite's ongoing P delivery, as the root system may respond locally to phosphate before it becomes soil-bound^46,47^. An important contribution to scavenging the soil for P is root extension^47^. Since the composites have a slower P release than the TSP treatment, the plant might have invested in a higher number of lateral roots for improved possibilities of scavenging for phosphate^48^. This should be further investigated in rhizotron experiments.

Consistent to leaf area results, fertilization with TPS was able to achieve a higher plant growth compared to the unfertilized control (Fig. 7). Although TPS shoot biomass was still statistically lower than that from P-fertilized treatments, plant height results were similar (Fig. 7a,b). Urea from the TPS structure could have had a role in sufficiently supplying nitrogen to support plant development (98.4 mg of N/pot), as it is one of the most required macronutrients for optimizing crop yields. Furthermore, the carbon content from TPS might promote the growth and activity of some heterotrophic microorganisms present in soils that can metabolize starch^24^. Thus, crops cultivated in soils with low levels of organic matter like sand may benefit from TPS from the composites. It is important to point out that organic acids generated from starch decomposition could also (i) acidify the medium and (ii) form complexes with metals, increasing phosphate availability and desorption in soils with high metal content^25^. The acidification from TPS biodegradation could contribute to struvite solubilization in St-TPS composites^24^.

Table 1 shows the available phosphate and final pH in the growth medium at the last day of cultivation. Triple superphosphate and pure thermoplastic starch showed the lowest and highest values of residual available phosphate in sand, with 11 mg/dm^3^ and 16 mg/dm^3^ respectively (Table 1). The lower residual P availability in sand from P-fertilized treatments is likely due to higher P uptake by the plants, as maize development was also higher in those treatments. Phosphorus assimilation in plants treated with only TPS or in the no fertilizer control was probably limited by the low availability of other nutrients, leading to a higher residual P in comparison. Moreover, residual phosphate concentration from slow-release fertilizers is usually higher at the vicinity of the fertilizer granules, a region where we observed an intense root growth, that could have interfered with the sand sampling. Therefore, residual available P from St and the composites in the sand samples could be underestimated.

**Table 1:** Average available phosphorus (as phosphate) and pH of the sand at the end of the greenhouse experiment, at 42 days of maize cultivation.

Treatment	P available (mg/dm^3^)	pH (CaCl_2_)
No fertilizer	13.0 ± 0.5 b	6.5 ± 0.2 cd
TPS	15.7 ± 2.0 a	6.7 ± 0.1 a
25St-TPS	12.9 ± 1.8 b	6.6 ± 0.1 bc
50St-TPS	11.8 ± 1.7 bc	6.5 ± 0.1 bd
75St-TPS	11.6 ± 1.1 bc	6.4 ± 0.2 d
St	13.0 ± 2.3 b	6.6 ± 0.1 ab
TSP	11.0 ± 0.9 c	6.6 ± 0.1 ab

Indexes a, b, c, and d represent the statistical differences between treatments (*p* < 0.05, n = 17).

As observed in the diffusion test, TSP is rapidly released in sand. Still, it did not outperform St-fertilizers regarding biomass production at the studied maize development stage. Thus, the results highlight that maize growth did not particularly benefit from TSP fast release in sand at the experiment duration. On the other hand, taking into account the slow-release behavior of struvite and the composites, it is possible that phosphate from these fertilizers was not completely delivered by the end of the pot experiment^30^. Nevertheless, maize yields proved struvite-P solubilization rate was sufficient to plant’s needs over the experimental duration. Therefore, P steady release from St and St-TPS proved to be favorable in highly permeable sand for maize cultivation.

Even though St-TPS composites featured initially a slower diffusion than pure St in the Petri dish test (Fig. 4), they achieved similar agronomic performances. Although it was not clear if the thermoplastic starch matrix can facilitate struvite-P dissolution with the particle dispersion effect, the results from this experiment suggest TPS could still have a valuable role, not only to avoid P leaching in field conditions but also as a carbon input with potential benefits to degraded soils. Nevertheless, more study is needed to confirm if starch decomposition indeed contributes to struvite solubilization and plant growth.

Substrate pH decreased in the presence of the fertilizers and maize (Table 1), being slightly lower in the presence of 75St-TPS (6.4) and higher with the pure TPS (6.7). The overall pH reduction is possibly from plant proton pump in response to nutrient uptake. It could also be related to root exudation of organic acids and carboxylates. Root biomass was highest in 75St-TPS and lowest in pure TPS, which possibly differently affected the pH by different amounts of root exudates. Another possible explanation would be the pH from the applied Hoagland solutions, as they were prepared with distinct nutrient contents for each treatment.

Struvite-thermoplastic starch composites and struvite displayed comparable performances to commercial TSP for maize growth, with higher biomass yields and plant height than unfertilized plants. In a pot experiment with maize, Giroto et al. (2020) found that NPK-thermoplastic starch composites of either TSP or Bayóvar rock achieved higher shoot biomass than when the P sources were directly mixed with the soil, i.e. not applied as composites^26^. Previous studies with struvite application reported various outcomes depending on the source of the recycled struvite and soil type, generally presenting an equal or even higher efficiency than water-soluble fertilizers. Similarly to the St-TPS composites, Cabeza et al. (2011) found a comparable effectiveness between recycled struvite and TSP in one year of maize cultivation, for both a neutral soil and an acid sand substrate^49^. In Liu et al. (2011), maize subjected to struvite featured similar plant height to a soluble reference in a sandy soil, but superior leaf area and biomass^50^. Likewise, in the work of Robles-Aguilar et al. (2020) maize cultivated in a sand substrate achieved significantly higher biomass and P uptake with struvite than TSP^51^. The same features were improved by struvite in relation to TSP and SSP in the studies by Vogel et al. (2015)^52^ and Nongqwenga et al. (2017)^53^.

## Conclusion

The present study focused on developing fertilizer composites containing ground struvite (St) distributed in a biodegradable and hydrophilic matrix of thermoplastic starch (TPS), using different proportions of the St and TPS. Struvite composites showed a P slow-release behavior in citric acid solution, with TPS functioning as a physical barrier to fast solubilization, indicating their suitability to be used in acidic environments such as in the plant root region, where protons and complexing agents exuded by plants are present. Phosphate diffusion investigated in neutral pH sand did not indicate that struvite dissolution was improved with its dispersion in TPS in this condition. However, the matrix showed a potential in regulating nutrient delivery and preventing nutrient losses. The possible agronomic benefits of the steady P-release from the composites were tested in maize plants. St-TPS composites featured a comparable performance to triple superphosphate (TSP) for projected leaf area and plant height, showing that phosphate release from St-TPS was sufficient to fulfill the plant demands. TPS alone appeared to contribute to plant growth when compared to the unfertilized control, likely attributed to its nitrogen content and carbon input. Struvite solubilization from the composites is assumed to benefit from TPS biodegradation and consequent organic acid release by soil microorganisms, which could be an advantage over the use of struvite only. Overall, St-TPS fertilizers demonstrated a capacity to simultaneously provide adequate P nutrition to maize in the highly permeable neutral sand, while reducing potential P leaching losses and environmental impacts observed in conventional fertilizers. Further studies should be conducted to test if TPS could in fact enhance struvite dissolution with its contribution to organic acid formation. Moreover, the agronomic efficiency of St-TPS composites should be tested under field conditions in an acidic soil, where struvite is rapidly solubilized and could benefit from the controlled-release provided by TPS, as indicated in the citric acid solution.

## Materials and methods

### Preparation of struvite-thermoplastic starch fertilizer composites

Composites consisting of struvite distributed in a TPS matrix were prepared to study the matrix effect on phosphate release. The composites contained three different percentages of struvite, i.e. 25, 50, and 75 wt%. A TPS material without struvite addition was also prepared to be used as one of the employed controls in the overall setup. The composites were prepared with corn starch (Amidex 3001—Ingredion, Brazil), urea (Yara, Brazil), and struvite (Ostara Crystal Green®, UK). Before composite preparation, struvite was pulverized using an orbital mill (Servitech, CT 241, Brazil) with alumina balls, followed by sieving (< 0.15 mm). Information on struvite composition can be found in the Supplementary Information (Table S1).

**Figure 8:**
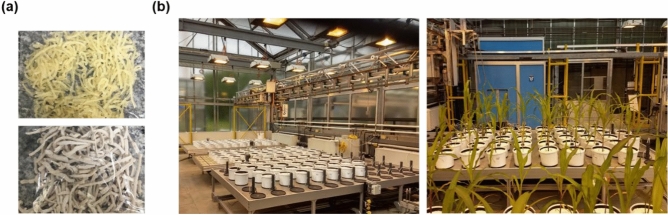
(**a**) Pure thermoplastic starch (top) and 75St-TPS composites (bottom), before grinding into smaller pieces. (**b**) The greenhouse experiment setup at the beginning of cultivation (left) and by the end (right).

Different masses of urea were added to each composite to achieve similar final N contents (wt%), functioning as a plasticizer to starch. First, urea was solubilized in water, followed by struvite powder addition under constant mixing by a mechanical stirrer. The amount of starch was then adjusted according to the intended struvite percentage of the final composite mass. The reaction was kept at 90 °C with a water bath until complete starch gelatinization. The gel materials were molded using a piping bag and placed in plastic trays, followed by drying at 50 °C in an oven overnight. The nomenclature of the fertilizers and nutrient contents are described in Table 2. Figure 8a shows the pure TPS material and the composite with 75 wt% struvite. Struvite-TPS composites and pure TPS were roughly cut (< 0.5 cm) before being characterized and tested.

**Table 2:** Nutrient contents (wt%) of N and P in struvite, urea, pure TPS, and the St-TPS composites, as well as the percentages (wt%) of struvite and urea added to TPS-based materials.

Materials	% Struvite	% Urea	% N	% P
Struvite	100.0	–	6.35	12.96
Urea	–	100.0	45.0	–
TPS	–	14.8	6.66	–
25St-TPS	25.0	10.1	6.28	3.02
50St-TPS	50.0	4.73	5.99	6.71
75St-TPS	75.0	0.36	6.47	10.98

### Materials characterization

The studied materials were analyzed by scanning electron microscopy (SEM) using a microscope (JEOL, JSM6510, Japan) with secondary electron mode. Prior to SEM analysis, samples were coated with a thin layer of gold in an ionization chamber (BalTec, Med. 020, Switzerland). Chemical structure elucidation was performed with Fourier-Transform Infrared (FTIR) analysis using a spectrometer (Brucker, VERTEX 70, Germany).

### P release under laboratory conditions

#### Phosphate and urea release in solution

A nutrient release experiment was conducted in beaker flasks filled with 500 mL of citric acid solution (2 wt%, pH = 2) to investigate phosphate and urea release behaviors from St-TPS composites over time^36^. The fertilizer dose was fixed as 400 mg of P per L of citric acid solution for St-TPS composites and a struvite reference. The struvite reference was used as received (1 mm granules). Detailed information on the initial urea concentrations used for St-TPS fertilizers and pure TPS can be found in Table S2.

The samples from the composites, pure TPS, and struvite were tested in triplicates. The flasks were kept in a chamber (Fanem, 347 CD, Brazil) with controlled temperature of 25 °C under constant agitation of 45 rpm. Aliquots for phosphate and urea quantification were collected every 24 h over eight days. Phosphate concentration was determined using an UV–Vis spectrophotometer (FEMTO, 700 Plus, Brazil), following the method from Murphy and Riley^54^. Urea release was simultaneously measured with a UV–Vis spectrophotometer (FEMTO, 700 Plus, Brazil) and estimated by an adapted method from Tomaszewska and Jarosiewicz^55^.

#### Phosphate diffusion experiment

Phosphate diffusion from the fertilizers was studied in a pH neutral sand (pH 7.3). Struvite-thermoplastic starch composites were compared to pure struvite and a commercial reference with high water solubility, i.e., triple superphosphate (TSP, Mosaic Fertilizantes, Brazil). The sand was selected as a model substrate to analyze the release dynamics without the interference of complex nutrient interactions with the growth medium or with plants, thus providing more reliable and reproducible results. The sand was obtained from an open gravel and sand pit in Kerpen-Buir, Germany, kindly provided by Rheinische Baustoffwerke GmbH, Germany. Before the test it was dried at room temperature and sieved (< 2 mm) to remove coarse particles. Detailed characterization of the sand can be seen in Table S3.

Based on the method by Degryse and McLaughlin^56^, Petri dishes (5.0 cm radius) were filled with 78 g of sand and wetted to 50% water holding capacity with deionized water, using triplicates of each treatment. The fertilizers were added after 24 h, positioned at the center of the Petri dish, and covered by the sand. A fixed-rate of 100 mg of P from the fertilizers per kg of sand was established, adjusted by adding different masses of each fertilizer. The Petri dishes were closed and kept in a chamber under controlled humidity and temperature of 25 °C. Phosphate visualization was conducted after 1, 8, 15, and 29 days following the methodology from Degryse and McLaughlin^56^. Briefly, hand-cut papers were first impregnated with Fe-oxide to capture phosphate. The wetted papers were placed on the sand substrate surface and pressed for either 10 min (for the Petri dishes incubated during one day) or 30 min (for the other incubation times). Phosphate diffusion zone was colored by modified malachite green. Images of the dry papers were recorded and analyzed with GIMP 2.10.14 software for diffusion area measurements.

### Greenhouse experiment

#### Experimental setup

The agronomic efficiency of St-TPS composites was investigated in a pot experiment with maize (*Zea mays*, ‘‘Badischer Gelber’’, Kiepenkerl-Bruno Nebelung GmbH, Germany), conducted from May to June 2019 in a greenhouse facility with controlled conditions at Forschungszentrum Jülich GmbH, IBG-2: Plant Sciences, Germany (50°54′36″N, 6°24′49″E). Plants received 16 h of light daily, in the form of natural and artificial light (≥ 400 µmol s^−1^ m^−2^, SON-T AGRO 400, Phillips), regulated by an automated light system. The average temperature and relative air humidity over the experiment time accounted for 24.1 °C and 46.75%, respectively. The greenhouse was equipped with an automatic shoot phenotyping platform, named “ScreenHouse”*,* to automatically record images of shoot development. Figure 8b shows the greenhouse set up at the beginning and the end of the cultivation period.

The fertilization effects of the struvite-thermoplastic starch composites (25St-TPS, 50St-TPS, and 75St-TPS) were compared to a negative control (no fertilizer), a positive reference of triple superphosphate (TSP), pure struvite (St), and pure thermoplastic starch (TPS). The pure TPS treatment was used as a reference, without additional nutrient supply. St-TPS composites, TSP, and St treatments were supplied with a fixed dose of 60 mg of P/pot (approximately 17.14 mg of P/dm^3^, equivalent to 34.82 kg of P/ha) and received additional pure TPS to equalize the amount of carbon to the value used in the pure TPS treatment (i.e., 572 mg of C/pot), as described in Table S4. A modified Hoagland solution was applied to St, St-TPS composites and TSP to complete the intended elemental concentrations of nitrogen (N, 127 mg/pot), potassium (K, 100 mg/pot), magnesium (Mg, 32.7 mg/pot), calcium (Ca, 21 mg/pot), and chloride (Cl, 2.9 mg/pot). The nutrient solution was prepared using stock solutions of (NH_4_)_2_SO_4_, KNO_3_, MgSO_4_·7H_2_O, K_2_SO_4_, and CaCl_2_·2H_2_O (Table S5).

The same sand used in the diffusion test was chosen as the growth medium for maize (Table S3). Prior to the experiment, the sand was dried at room temperature and sieved (< 2 mm). Pots (3.5 L) were filled with a total of 4.15 kg of sand. First, 2.15 kg of sand were added into the pots and the fertilizers were placed at the center. Following this, the fertilizers were covered with 2 kg of sand, mimicking below-ground field application, inspired by the CULTAN method. A total of 17 replicates was used for each treatment, accounting for 119 pots in total.

Maize seeds were pre-germinated separately for one week in multi-well plates containing the same sand substrate. Only morphologically comparable seedlings were transplanted thereafter. One seedling was transplanted to the center of each pot above the fertilizer, at a 2 cm depth from the sand surface, followed by watering with 500 mL of rainwater. After two days, the substrate surface was covered with white polypropylene beads (170 g/pot) to reduce water evaporation and to maintain an appropriate contrast for the shoot image analysis. Modified Hoagland solution (200 mL) was applied to the P-fertilized treatments (St, 25St-TPS, 50St-TPS, 75St-TPS, and TSP) 16 and 30 days after transplanting the seedlings, with different compositions for each treatment as indicated in Table S5. Pots were watered automatically by the “ScreenHouse” platform after shoot area measurements (see below) to maintain the humidity of sand at 50% of its water holding capacity. Pots were automatically randomized by the system during the measurements to avoid edge and microclimate effects.

#### Non-invasive measurements

To observe the shoot growth dynamics, which varies according to nutrient availability, shoot images of maize plants were non-invasively recorded in the “ScreenHouse” platform (Fig. 8b) twice a week as described in Herzel et al.^57^. Briefly, plant images were recorded automatically from a 45° camera angle (Point Gray Grasshopper2, 5 MP color camera, FLIR Integrated Imaging Solutions Inc., Richmond, BC, Canada), using a rotating table to allow images from four sides, i.e. 0°, 90°, 180°, and 270° rotation of the pots. Shoot images were analyzed to estimate the projected leaf area and the percentage of brownish area on the leaves, corresponding to nutrient deficiency. Color HSV-segmentation masks for green and brownish regions were obtained using an in-house developed software from the IBG-2: Plant Sciences, Forschungszentrum Jülich GmbH. The software was part of the toolbox, earlier described in Müller-Linow et al.^58^. Projected leaf area results are shown in pixels (px)^59^.

Plant growth was also analyzed based on plant height, which was measured by hand on the harvest day, i.e., 42 days after transplanting.

#### Post-harvest biomass and sand analysis

Shoots and roots were separated and dried in a forced-draft oven at 65 °C, followed by dry weight measurements. The sand substrate was air-dried and analyzed to estimate the concentration of remaining available nutrients. Plant available phosphate was extracted from the substrate with water and anionic resin, followed by the determination of phosphate concentration with UV–Vis spectrophotometer (FEMTO, 600 Plus, Brazil), according to Quaggio and Raij^60^. The growth medium pH was measured after mixing 10 cm^3^ of the sand samples in 25 mL of CaCl_2_ solution (0.01 mol/L), using a pH meter (Micronal, B474, Brazil).

### Statistical Analysis

Statistical analysis was performed using one-way analysis of variance (ANOVA) at the significance level *p* < 0.05 (Origin Pro 9.0, USA), with mean comparisons by Tukey's test, homogeneity of variance by Levene's test, and power analysis. Treatments were compared in the phosphate diffusion experiment (effective radius) and at the end of the greenhouse experiment (final plant height, shoot and root dry weights, projected leaf area, and sand pH, available phosphate and magnesium).

We confirm that all methods and research on plants were performed in accordance with the relevant guidelines and regulations.

## Supplementary Information

Supplementary Information.

## Data Availability

All data generated or analyzed during this study are included in this published article and its supplementary information file.
