# Supplementary material for: Struvite-based composites for slow-release fertilization: a case study in sand
Source: Sci Rep. 2022 Aug 19;12:14176. doi: 10.1038/s41598-022-18214-8 (PMC9391495; doi:10.1038/s41598-022-18214-8)
Supplement: Supplementary file 1 — Supplementary Information. [file 41598_2022_18214_MOESM1_ESM.pdf]

Supplementary Information

Struvite-based Composites for Slow-Release Fertilization: A Case Study in Sand

Stella F. Valle [a,b], Amanda S. Giroto [b], Vitalij Dombinov [c], Ana A. RoblesAguilar [d], Nicolai D. Jablonowski [c], Caue Ribeiro [b]*.*

[a] Federal University of São Carlos, Department of Chemistry, Washington Luiz Highway, km 235, 13565-905, São Carlos, SP, Brazil.

[b] Embrapa Instrumentation, XV de Novembro Street, n 1452, 13560-970, São Carlos, SP, Brazil.

[c] Forschungszentrum Jülich GmbH, Institute of Bio- and Geosciences, IBG-2: Plant Science, 52425, Jülich, Germany.

[d] Ghent University, Faculty of Bioscience Engineering, Department of Green Chemistry and Technology, Campus Coupure, Infinity House, Coupure Links 615, 9000 Ghent, Belgium.

*email: n.d.jablonowski@fz-juelich.de; caue.ribeiro@embrapa.br.

Table of Contents

I) Materials and Methods....................page 3

Struvite Characterization....................page 3

Phosphate and Urea Release in Solution....................page 3

Sandy Substrate Characterization....................page 4

Greenhouse Experiment - Nutrient Supply Details....................page 5

II) Results....................page 6

Urea Release Trends in Citric Acid Solution....................page 6

Greenhouse Experiment – Projected Leaf Area and Brown Leaf Area Over Time....................page 8

Greenhouse Experiment - Shoot:Root-ratio....................page 9

References....................page 9

I) **Materials and Methods**

Struvite Characterization

Table S1: Elemental composition (wt.%) of struvite, determined with X-ray fluorescence (Malvern Panalytical, MiniPal 4, UK) and CHN elemental analysis (Perkin Elmer, 2400, USA).

Element	wt.%
P	12.96
Mg	7.07
Ca	1.84
Al	0.56
N	6.35
C	0.32

Phosphate and Urea Release in Solution

Table S2: Phosphorus concentration used in the release test in citric acid solution (2 wt.%), and corresponding initial urea concentration from each material (mg/L of solution).

Materials	mg of P/L	mg of Urea/L
Struvite	400	-
TPS	-	1660
25 St_TPS	400	1337
50 St_TPS	400	284
75 St_TPS	400	13

Sandy Substrate Characterization

Table S3: Characterization of the sandy substrate used in the Petri dish test and the greenhouse pot experiment. Available nitrogen (ammonium and nitrate), phosphorus, potassium, and magnesium were extracted with $CaCl_2$/DTPA (CAT).

Parameter	Unit	Value
dry substance	%	92.9
bulk density (wet)	g/L	1285
bulk density (dry)	g/L	1195
pH (in CaCl)		7.3
conductivity (in H_2O)	µS/cm	19
salt (as KCl in H_2O)	g/L	0.13
salt (as KCl in $CaSO_4$)	g/L	< 0.10
nitrogen (mineral)	mg/L	2
ammonium-N	mg/L	< 1
nitrate-N	mg/L	2
phosphorus (P_2O_5)	mg/L	< 2
potassium (K_2O)	mg/L	< 4
magnesium	mg/L	32

Greenhouse Experiment - Nutrient Supply Details

Table S4. Treatments regarding fertilizer supply, additional TPS, and nutrient solution application. The mass of fertilizer added to each treatment corresponds to 60 mg of P/plant, and additional TPS was applied to complete 572 mg of C/plant.

Treatment	Fertilizer (g)	Additional TPS (g)	Nutrient solution
No Fertilizer	-	-	no
TPS	-	1.48	no
25 St_TPS	1.99	-	yes
50 St_TPS	0.89	0.98	yes
75 St_TPS	0.55	1.32	yes
St	0.46	1.48	yes
TSP	0.31	1.48	yes

Table S5. Volume of 1M stock solutions (mL) used to prepare 1 L of modified Hoagland solution (1/3 strength).

Treatments	Volume of 1M stock solution (mL)				
	$(NH_4)_2SO_4$	KNO_3	$MgSO_4{\cdot}7H_2O$	K_2SO_4	$CaCl_2{\cdot}2H_2O$
No Fertilizer	-	-	-	-	-
TPS	-	-	-	-	-
25 St_TPS	0.30	0.4	3.0 (1/3)	0.5 (1/3)	5.9
50 St_TPS	1.0	1.1	1.4 (1/3)	5.7 (1/3)	0.7
75 St_TPS	0.5	0.5	1.0 (1/3)	6	1
Struvite	-	-	-	6.2	1.1
TSP	3.5	4	6.6 (1/3)	4.2	2

II) Results

Urea Release Trends in Citric Acid Solution

Urea release patterns from St_TPS composites and pure TPS were monitored simultaneously to phosphate release in citric acid solution (**Figure S1**). It is important to point out that the applied urea concentration varied with each material (**Table S2**), as the amount of fertilizer added to the solution was fixed by the P concentration.

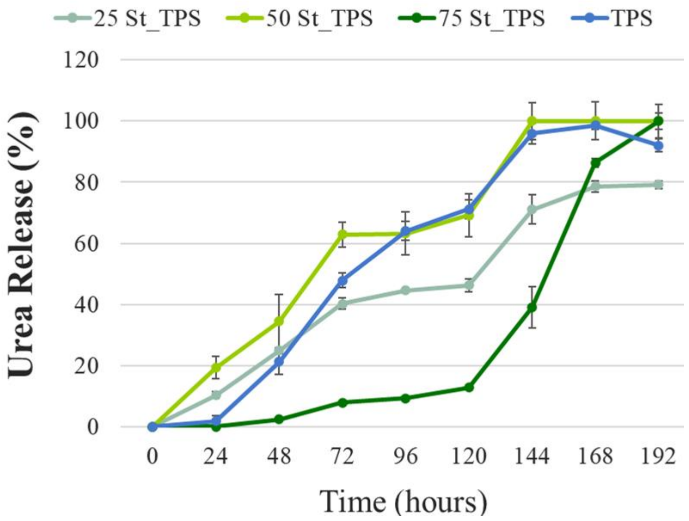

Figure S1: Urea release trends in citric acid solution (2 wt.%) at 25ºC and pH 2. Points show mean values ± standard deviations (n=3).

Urea solubilization in citric acid is known to occur within only 2 to 3 hours.[1,2] Contrary to the fast solubilization of pure urea, a delayed-release was observed in the fertilizer composites (**Figure S1**). Besides the matrix hindering effect as a physical barrier, urea strongly interacts with the polymer chains as a plasticizer to thermoplastic starch structure, contributing to the slower delivery rate observed in all TPS materials.

Urea release from 50 St_TPS was slightly faster than pure TPS after the first 72 hours, which could be related to struvite presence disrupting the intensity of urea-starch interactions, both chemically and physically. 75 St_TPS behaved differently to the other materials, maintaining a slower rate during the first 125 hours, after which urea delivery proceeded rapidly. In this case, struvite mass content in the composite was superior to TPS mass, encapsulating the polymer. **Figure 4** showed that after 125 hours, around 40% of phosphate from 75 St_TPS was released to the solution, probably resulting in increased exposure of TPS and hence in the observed boost of urea delivery. Complete urea solubilization was only reached after 175 hours, except for 25 St_TPS, which released only 80%. The applied urea concentration from 25 St_TPS was significantly superior to the other composites, around 5 and 103 times the values from 50 St_TPS and 75 St_TPS, respectively. Therefore, it is possible that the solution reached its saturation, affecting the dynamic of dissolution and nutrient release.

Greenhouse Experiment – Projected Leaf Area and Brown Leaf Area Over Time

Table S6. Average projected leaf area (px) and estimated brown leaf area (%) over the time of maize cultivation. Indexes a, b, and c represent the statistical differences between treatments at each time, with $p < 0.05$ (n=16 for total leaf area; n=13 for brown leaf area).

	**14 days**	**21 days**	**28 days**	**35 days**	**41 days**
**Treatment**	**Projected Leaf Area (10^3 px)**				
No Fertilizer	12.1 ± 2.5 c	19.7 ± 7.5 c	31.3 ± 20.1 c	41.8 ± 36.0 d	53.1 ± 45.1 c
TPS	18.3 ± 5.1 b	36.5 ± 12.2 b	72.2 ± 27.0 b	127.3 ± 40.8 bc	187.4 ± 40.9 b
25St-TPS	19.7 ± 6.4 ab	40.2 ± 15.6 ab	89.7 ± 32.7 ab	177.3 ± 55.9 a	254.8 ± 52.8 a
50St-TPS	20.5 ± 5.1 ab	41.3 ± 13.3 ab	89.2 ± 31.6 ab	187.6 ± 49.2 a	262.6 ± 56.9 a
75St-TPS	24.0 ± 5.1 a	53.3 ± 17.8 a	108.8 ± 28.7 a	212.7 ± 49.5 a	289.3 ± 42.4 a
St	22.9 ± 3.9 ab	45.1 ± 7.6 ab	97.5 ± 14.1 ab	197.4 ± 24.3 a	278.4 ± 35.0 a
TSP	20.9 ± 4.3 ab	39.9 ± 6.9 b	84.5 ± 20.2 ab	171.0 ± 41.7 ac	252.2 ± 59.3 a
**Treatment**	**Brown Leaf Area (%)**				
No Fertilizer	2.9 ± 1.2 ab	10.6 ± 3.8 a	13.6 ± 6.2 a	28.5 ± 15.3 a	25.4 ± 11.9 a
TPS	4.4 ± 3.0 a	7.5 ± 3.7 ab	7.4 ± 2.6 b	8.5 ± 3.4 b	8.2 ± 2.5 b
25St-TPS	2.5 ± 2.0 abc	5.4 ± 4.0 bc	3.9 ± 2.4 b	4.5 ± 1.7 b	6.2 ± 2.4 b
50St-TPS	0.7 ± 0.4 bc	5.7 ± 3.6 bc	4.9 ± 2.4 b	4.2 ± 1.4 b	6.4 ± 1.4 b
75St-TPS	0.6 ± 0.5 c	3.6 ± 2.5 c	4.0 ± 2.3 b	5.4 ± 2.2 b	7.2 ± 2.3 b
St	2.4 ± 2.3 abc	5.2 ± 1.5 bc	3.5 ± 1.2 b	4.3 ± 1.7 b	6.7 ± 1.5 b
TSP	1.4 ± 1.2 bc	3.9 ± 1.8 bc	5.2 ± 2.8 b	5.0 ± 2.3 b	5.8 ± 1.1 b

Greenhouse Experiment - Shoot:Root-ratio

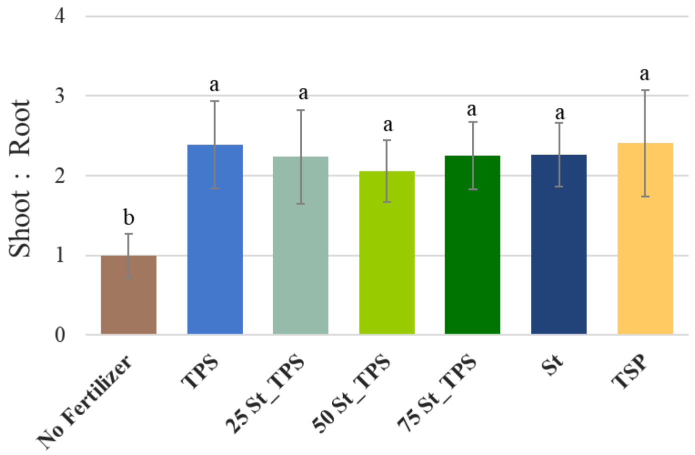

Figure S2: Shoot:root-ratio of each treatment. Bars show mean values ± standard deviations (n = 16). Indexes a and b identify significant statistical differences between treatments ($p < 0.05$).

References

1. Giroto, A. S., Fidélis, S. C. & Ribeiro, C. Controlled release from hydroxyapatite nanoparticles incorporated into biodegradable, soluble host matrixes. *RSC Adv.* **5**, 104179–104186 (2015).

2. Giroto, A. S., Guimarães, G. G. F., Foschini, M. & Ribeiro, C. Role of Slow-Release Nanocomposite Fertilizers on Nitrogen and Phosphate Availability in Soil. *Sci. Rep.* **7**, 46032 (2017).
